# A stroke rehabilitation training program for community-based primary health care, South Africa

**DOI:** 10.4102/ajod.v12i0.1135

**Published:** 2023-03-06

**Authors:** Elsje Scheffler, Robert Mash

**Affiliations:** 1 Division of Family Medicine and Primary Care, Faculty of Medicine and Health Sciences, Stellenbosch University, Cape Town, South Africa; 2 Centre for Disability and Rehabilitation Studies, Faculty of Medicine and Health Sciences, Stellenbosch University, Cape Town, South Africa

**Keywords:** Primary healthcare, family caregiving, stroke rehabilitation, community care, education and training, participative methods

## Abstract

**Background:**

With an increasing burden of stroke and a lack of access to rehabilitation services in rural South African settings, stroke survivors rely on untrained family caregivers for support and care. Community health workers (CHWs) support these families but have no stroke-specific training.

**Objectives:**

To describe the development of a contextually appropriate stroke training program for CHWs in the Cape Winelands District, South Africa.

**Method:**

Twenty-six health professionals and CHWs from the local primary healthcare services participated in action research over a 15-month period from September 2014 to December 2015. The groups participated in two parallel cooperative inquiry (CI) groups. The inquiry followed the cyclical steps of planning, action, observation and reflection. In this article, the planning step and how the CI groups used the first three steps of the analyse, design, develop, implement, evaluate (ADDIE) instructional design model are described.

**Results:**

The CHWs’ scope of practice, learning needs, competencies and characteristics, as well as the needs of the caregivers and stroke survivors, were identified in the analysis step. The program design consisted of 16 sessions to be delivered over 20 h. Program resources were developed with appropriate technology, language and instructional methodology.

**Conclusion:**

The program aims to equip CHWs to support family caregivers and stroke survivors in their homes as part of their generalist scope of practice. The implementation and initial evaluation will be described in a future article.

**Contribution:**

The study developed a unique training program for CHWs to support caregivers and stroke survivors in a resource-constrained, rural, middle-income country setting.

## Introduction

South Africa (SA) and other low- and middle-income countries (LMICs) have an increasing burden of stroke as a result of socio-economic development, urbanisation, increasing risk factors for noncommunicable diseases and a transition in the epidemiological profile of the population associated with ageing, increase in cardiovascular diseases and adoption of the Western lifestyle (Donkor 2018; Langhorne et al. 2018; Taylor & Ntusi 2019). While significant functional improvement is possible, around a third of stroke survivors require ongoing care (Jaracz et al. 2015). In rural SA, the incidence and prevalence of stroke are higher than elsewhere recorded in SA or other African countries (Maredza, Bertram & Tollman 2015; The SASPI Project Team 2004), and the lack of access to medical and rehabilitation services increases disability and impairment, with resultant dependence on family caregivers. While family caregiver training is integral to stroke care and rehabilitation in all settings and levels of the health system (Cameron et al. 2016; Lindsay et al. 2014; Pandian et al. 2016; Winstein et al. 2016), the lack of services in rural SA contexts means a lack of caregiver training in these communities.

The local and international literature describes a myriad of caregiver training interventions in different levels of care, different settings and with different caregiver needs and stroke survivor stages of recovery (Aslani et al. 2016; Bakas et al. 2014; Brereton, Carroll & Barnston 2007; Camak 2015; Chaiyawat & Kulkantrakorn 2012; Chaiyawat, Kulkantrakorn & Sritipsukho 2009; Deyhoul et al. 2020; Forster et al. 2013; Hafsteinsdóttir et al. 2011; Krieger, Feron & Dorant 2017; Lutz et al. 2016; Mudzi, Stewart & Musenge 2012; Nordin et al. 2014; Pitthayapong et al. 2017; Robinson et al. 2005; The ATTEND Collaborative Group 2017; Smith et al. 2004; Torres-Arreola et al. 2009; Wang et al. 2015; Yan et al. 2016; Zhou et al. 2019). Most evidence, including evidence from SA, comes from stroke survivors who received specialised stroke unit care and/or multidisciplinary rehabilitation services (Aslani et al. 2016; Bakas et al. 2014; Brereton et al. 2007; Camak 2015; Dobe, Gustafsson & Walder 2022; Forster et al. 2013; Hafsteinsdóttir et al. 2011; Krieger et al. 2017, 2021; Lutz et al. 2016; Robinson et al. 2005). There is limited evidence for caregiver training interventions in settings where these services are not available (Deyhoul et al. 2020; Dobe et al. 2022; Krieger et al. 2021; Mudzi et al. 2012; Nordin et al. 2014; Torres-Arreola et al. 2009; Zhou et al. 2019). Despite the volume of evidence on caregiver training, few publications detail the training development process (Forster et al. 2015; Krieger et al. 2017; Robinson et al. 2005). This inadequate description of the development process, particularly the context and instructional design methods, limits the transferability of interventions to low-resourced primary healthcare (PHC) settings. There is also no evidence of caregiver training as a PHC intervention.

South African health policy promotes a PHC approach with a continuum of preventive, curative, rehabilitative and palliative services. Home- and community-based care (HCBC) are delivered by nurse-led teams of community health workers^1^ (CHWs) (Naledi, Barron & Schneider 2011; National Department of Health 2018). Rehabilitation services at the PHC level are limited and infrequent (Rhoda, Mpofu & De Weerdt 2009) and often inaccessible because of poverty and a lack of or inaccessible transport (Cawood & Visagie 2016; Eide et al. 2015; Maart et al. 2007; Maart & Jelsma 2014; Visagie & Swartz 2016).

National initiatives to strengthen PHC rehabilitation services include strengthening the role of CHWs, as well as introducing midlevel rehabilitation workers^2^ (National Planning Commission 2012). However, the latter has not gone to scale, and there is a need to redress the inequitable distribution of rehabilitation services. Stroke caregiver training is congruent with the scope of practice of CHWs (Hartzler et al. 2018). Unfortunately, the national CHW training curriculum contains limited rehabilitation content and only as elective modules (South African Qualifications Authority 2018a, 2018b, 2018c), thus limiting the knowledge of CHWs to support family caregivers.

**FIGURE 1:**
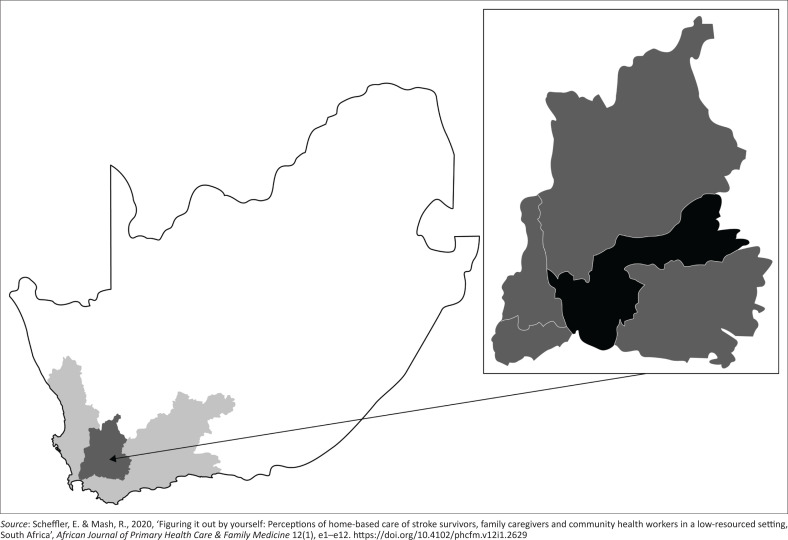
Map of South Africa illustrating the Western Cape province (light grey area), with the Cape Winelands district (darker grey area). The insert shows the Breede Valley subdistrict (black).

The Western Cape province in SA (Figure 1) has a higher prevalence of stroke compared with national figures (Shisana et al. 2013; Statistics South Africa 2018a). Within its less-resourced rural districts, there are no stroke units or inpatient rehabilitation services. Acute inpatient stays are an average of five days (Scheffler & Mash 2019), and stroke survivors are discharged home to untrained family caregivers, resulting in poor stroke survivor and caregiver outcomes and satisfaction (Scheffler & Mash 2019) and a high caregiver burden (Scheffler & Mash 2020). For these stroke survivors and their family caregivers, CHWs may be their only accessible source of health care and rehabilitation. However, these CHWs have no stroke or rehabilitation-specific training. Because of the lack of rehabilitation service capacity in the district, a community-based services (CBS) manager from the rural Cape Winelands District (Figure 1) requested the researcher’s assistance with the development of a rehabilitation training program for CHWs to train family caregivers of stroke survivors within HCBC services. The district had one multidisciplinary professional rehabilitation team of seven members and no midlevel rehabilitation workers. The researcher, a physiotherapist, had extensive stroke rehabilitation experience in low-resourced settings and with designing and developing training programs for rehabilitation health workers. This article reports on the design and development of a contextually appropriate stroke training program for CHWs within a rural South African PHC context.

## Research methods and design

This article reports on the last stage of a multistage mixed-methods study (Table 1). The first two convergent stages, a quantitative longitudinal survey (Scheffler & Mash 2019) and a qualitative exploratory descriptive study (Scheffler & Mash 2020), preceded the participatory action research study in stage three.

**TABLE 1:** Overview of the multistage study, procedures and results integrated with the steps of the ADDIE model.

Study stage	Steps of the ADDIE model	Procedures		Products
Stage 1	Analyse	Longitudinal survey (Scheffler & Mash 2019)		Demographic and socio-economic profile, including living conditions and education of stroke survivors Function and independence of stroke survivors Caregiver strain Stroke survivor and caregiver satisfaction with services Acute hospital and primary care services received
Stage 2	Analyse	Qualitative descriptive exploratory study (Scheffler & Mash 2020)		Perceived needs of stroke survivors, caregivers, and community health workers
Stage 3	Analyse	Participatory action research using cooperative inquiry	Planning	Design and develop training program and resources
	Design			
	Develop			
	Implement		Action	Pilot training program and evaluate outcomes
	Evaluate		Observation	
			Reflection	

ADDIE, analyse, design, develop, implement, evaluate.

In this last stage of this multistage mixed-methods study, a cooperative inquiry (CI) process was used (Heron 2014; Higginbottom & Liamputtong 2015; Ramsden et al. 2018). Cooperative inquiry had been used in stroke program and training development (Aslani et al. 2016; Dobe et al. 2022; Krieger et al. 2021). The CI followed the cyclical steps of planning, action, observation and reflection (Table 1). The cyclical steps of the CI were also aligned with the analyse, design, develop, implement, evaluate (ADDIE) (analyse, design, develop, implement, evaluate) instructional design model (Allen 2006; Mayfield 2011) for the development of a training program (Table 1): analyse, design, develop, implement and evaluate.

**FIGURE 2:**
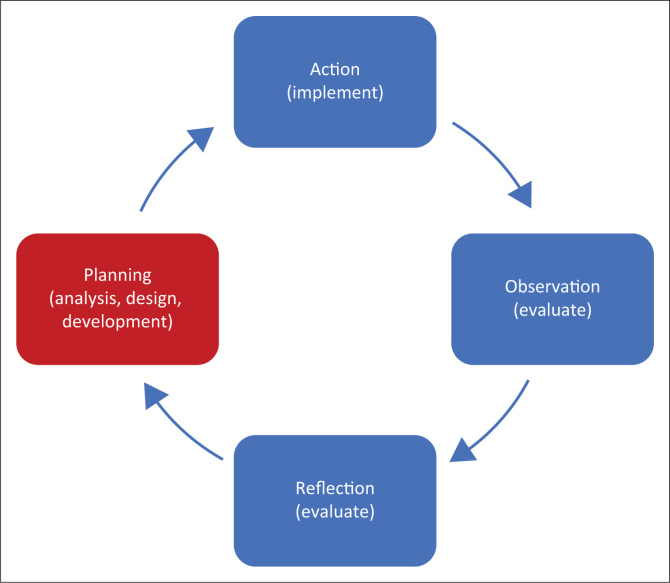
Diagrammatic representation of the methods. The cycle illustrates the 4 steps of the cooperative inquiry (planning, action, observation and reflection) with the relevant steps of the analyse, design, develop, implement, evaluate model in brackets. This article focuses on the planning step of the cooperative inquiry.

This article focuses only on the planning step of the CI, which corresponds with the analysis, design and development steps of the ADDIE model (Figure 2). A separate article will report on the remaining steps of the inquiry where the program, per the ADDIE model, was implemented and evaluated.

### Setting

The study was conducted in the Breede Valley Subdistrict (Figure 1) of the Cape Winelands District in the Western Cape, South Africa. In this rural setting, the majority of the population of 866 000 live in poverty and are dependent on public sector services (Statistics South Africa 2018b). The predominant language in the district is Afrikaans, followed by English and isiXhosa.

Stroke clinical practice pathways, evidence-based practice guidelines, as well as referral guidelines, were absent on the HCBC platform, and stroke care was poorly coordinated (Scheffler & Mash 2019). Stroke survivors were discharged home from acute care to untrained family caregivers. Function and care were limited by numerous environmental barriers such as unavailable or inaccessible services, physical barriers and lack of assistive products (Scheffler & Mash 2019, 2020a). Overall literacy and numeracy of both stroke survivors and caregivers were low, with more than half having no or only primary school education (Scheffler & Mash 2019, 2020a). Knowledge of stroke, rehabilitation, services and assistive products was poor (Scheffler & Mash 2019, 2020a).

Four independent organisations delivered rehabilitation-related services to stroke survivors and their families free of charge:

- Seven healthcare professionals, all from different professions, referred to as the multidisciplinary professionals (MDPs) (Table 2) from the Department of Health rotated through the primary care facilities and delivered individual rehabilitation services. Therapists, on request of HCBC services, had previously provided short, informal *ad hoc* in-service training sessions to CHWs.
- Boland Hospice, a nonprofit organisation, was funded by the Department of Health to deliver HCBC services in 10 municipal wards. Four home-based care coordinators, who were professional and enrolled nurses, were responsible for conducting assessments, designing treatment plans and supervising the 79 CHWs (Table 3) delivering home-based care services.
- The Breede Valley Association for the Physically Disabled (APD), a nonprofit organisation, provided a range of services for persons with disabilities, including stroke survivors, such as access to education, employment and therapeutic counselling services.
- Undergraduate physiotherapy and speech therapy students from Stellenbosch University (SU)’s Rural Clinical School were placed in two of the municipal wards and often accompanied the CHWs. They were supervised by two physiotherapists and a speech therapist from the university.

**TABLE 2:** Numbers of multidisciplinary professionals and community health workers in the Breede Valley subdistrict.

Profession	*n*
Physiotherapists	1
Occupational therapists	1
Speech therapists	1
Social workers	1
Oral hygienists	1
Clinical psychologists	1
Dieticians	1
Community health workers	79

**TABLE 3:** Participant profile of the two cooperative inquiry groups.

Characteristics	Community-based services CIG			Multidisciplinary professionals CIG	
	Community-based service management	Boland Hospice	Association for physically disabled	District	University
Total participants	3	11	4	5	3
**Professions**					
Operational manager	2†	1	1†	0	0
Nurse (enrolled or professional)	1	4	0	0	0
Social worker	0	1	2	1	0
Occupational therapist	0	0	1	1	1
Physiotherapist	0	0	0	1	1
Oral hygienist	0	0	0	1	0
Speech therapist	0	0	0	1	1
Community health worker	0	5	0	0	0
**Gender**					
Male	0			1	
Female	18			7	
**Age range**					
20–29 years	0			2	
30–39 years	7			2	
40–49 years	8			3	
50–59 years	2			1	
60+ years	1			0	
**Time in profession**					
1–5 year	4			1	
6–10 years	5			4	
11–20 years	6			1	
21+ years	3			2	

CIG, cooperative inquiry group.

† , Three of the managers were trained professionals: a social worker, professional nurse and occupational therapist.

### Selection of cooperative inquiry group members

The researcher consulted with the respective service managers from all the groups described in the setting section to identify key role players. Because of practical and logistical considerations such as transport problems and work schedules, two cooperative inquiry groups (CIGs) were formed (Table 3). All but one invited person joined the process.

The CBS-CIG consisted of 18 service providers from the Cape Winelands District CBS management, Boland Hospice and APD. The MDP-CIG consisted of eight MDPs from the Breede Valley District and the university and included both clinical and educational expertise.

### The inquiry process

Both CIGs engaged in a 15-month collaborative inquiry from September 2014 to December 2015. The inquiry focused on the interprofessional design and development of an appropriate training program for CHWs to train family caregivers of stroke survivors. Cooperative inquiry group members contributed from their own experience, knowledge and available evidence related to caregiver training needs and interventions. The researcher, who was ultimately responsible for driving the research agenda and processes, engaged in a consultative and collaborative manner (Higginbottom & Liamputtong 2015) and shared findings and conclusions between the two CIGs. A mutually designed timetable and activity chart guided the process. This, together with small group assignments, contributed to accountability. With the diversity in members of the CBS-CIG, a dedicated effort was made by the researcher to create a neutral, democratic and collaborative meeting space. The inquiry process is described under the first three steps of the ADDIE model.

#### Analysis step

During this step, the CIGs triangulated findings from the preceding studies (Scheffler & Mash 2019, 2020a), the available evidence (Cameron et al. 2016; Forster et al. 2012; Lindsay et al. 2014) and their own professional experience to define the CHWs’ learning needs and characteristics as learners, taking into account the CHWs’ broader scope of practice. They also considered the needs of potential trainers, their characteristics as educators, as well as the training resources required. Finally, they analysed the community and service context within which CHWs would need to perform after training.

#### Design step

In the design step, the CIGs utilised the information generated in the analysis step to formulate the following:

- learning outcomes
- educational approach
- rehabilitation philosophy
- training resources required
- approach to assessment of trainees
- structure of the training and time allocation.

#### Development step

In this phase, the CIGs developed learner and trainer materials and resources, as well as learner assessment tools.

### Data collection and consensus building

Data collection and consensus building are described under the first three steps of the ADDIE model.

#### Analysis step

Both CIGs held three 3-h meetings and participated in group discussions and nominal group activities to reach consensus and rank ideas (McKillip 1987). All meetings were audio-recorded and transcribed. Small group activities were recorded using field notes, flipchart summaries and photographs. The researcher compiled a detailed summary of each meeting, which was validated at the next meeting.

#### Design step

Using the same activities, both CIGs met twice to reach a consensus on the design. Following the development and validation of the key programmatic and sessional learning outcomes, the researcher developed an outline of the course structure with key topics, which were reviewed and revised by the CIGs via e-mail. Following consensus on the structure and time allocation, the final design was critically reviewed by five external reviewers who were experts in the field of stroke rehabilitation and training or education.

#### Development step

The researcher was primarily responsible for the development of the materials according to the design and collaborated closely with relevant CIG members. The psychosocial management session was developed by a CIG subgroup of four social workers and one occupational therapist. The materials were developed according to the design, with attention to evidence-based rehabilitation practice and what was feasible in the local context. Consensus building and validation within the CIGs followed the same process as described in the design step above. All CIG members and the external reviewers reviewed the final materials.

### Ethical considerations

The study was approved by the Health Research Ethics Committee 1 at Stellenbosch University (ref. no. S13/09/158), and permission was obtained from the Department of Health to conduct the study.

## Results

This section reports on the results of the planning of the CIGs in the first three steps of the ADDIE model, namely analyse, design and develop.

### Analysis

The following key factors that influenced the design of the training course were identified.

#### Community health workers’ scope of practice

The CHWs did not have any stroke- or rehabilitation-specific training. Their scope of practice already included health education, basic observations, training of family caregivers, assistance with daily living, monitoring patient and caregiver function and well-being, monitoring of treatment adherence, identification and referral of psychosocial problems and excluded patient assessment and care plan development (Boland Hospice 2014).

As generalists on the PHC platform, their prior training incorporated key principles of person-centeredness, continuity of care and intersectoral coordination. These principles were integrated into the new learning materials while taking note of existing competencies. Care was taken not to duplicate the scope of practice of midlevel rehabilitation workers.

#### Community health workers’ learning needs

The consensus on the CHW learning needs was positioned within their scope of practice and included:

- knowledge about stroke: what it is, risk factors, causes, prevention, recovery
- knowledge about stroke rehabilitation: rehabilitation services available in the province, district and HCBC and the roles and responsibilities of CHWs, caregivers and patients
- knowledge to provide emotional support, reduce and monitor caregiver strain and identify psychosocial problems
- knowledge and skills to teach caregivers how to assist stroke survivors with basic activities of daily living, positioning, transfers and mobility
- knowledge on assistive products, including low-cost alternatives and how to access these
- knowledge on stroke survivor and caregiver safety, including prevention of falls and prevention of secondary complications
- ability to problem-solve around common environmental barriers
- knowledge and skills to teach basic rehabilitation exercises.

Distinct differences, however, existed between the two CIGs on how these learning needs were phrased. Whereas the CBS-CIG formulated learning needs in terms of plain language and functional activities, the MDP-CIG used professional jargon and emphasised impairments and theoretical principles. The final consensus was to formulate learning needs in terms of practical and functional activities that were informed by underlying theory, even if the theory was not always explicit.

#### Community health worker competencies

Six key CHW competencies needed to train caregivers were identified. Although these competencies all existed in a generic way, they needed to be specifically related to stroke:

- transfer knowledge to caregivers and stroke survivors by explaining, informing and educating
- transfer skills to caregivers and stroke survivors through explanation, demonstration and practice with feedback
- evaluation of caregivers’ skills and knowledge
- provide emotional support to caregivers and stroke survivors and monitor for caregiver strain
- know when and how to get help from medical, rehabilitation and social services
- manage themselves in terms of their own roles and boundaries, as well as caregivers’ and stroke survivors’ expectations.

#### Characteristics of community health workers as learners

Although the CHWs had all completed high school, the majority only had basic literacy and numeracy competencies. The first language of the majority was Afrikaans, followed by isiXhosa and English, with English being the common language. In addition to in-service training, some had completed basic education modules in home-based care. They preferred learning skills through observation, role-play and practice with feedback. Although they had limited knowledge, the CHWs were highly receptive to training on home-based stroke rehabilitation and motivated to learn more.

#### Characteristics of the trainers

Training was to be delivered by the MDPs, who had experience in stroke rehabilitation and working in low-resourced settings. Although having previously provided in-service training, they had no background in the development of formal structured training programs targeting a community-based health problem.

#### Trainer resources required

No appropriate existing training materials could be identified. Both CHWs and caregivers complained about having received conflicting information in the past, highlighting the need for a trainer’s manual with detailed session plans to ensure a structured, uniform training approach and content. This would be an important resource for use by MDPs and their students.

#### Service contextual factors

The lack of clinical practice pathways at the HCBC level resulted in a poor understanding of the CHWs’ rehabilitation role, as well as unmet expectations and unrealistic demands of stroke survivors and caregivers. These factors, together with a lack of insight and support from formal services, resulted in many CHWs assuming additional tasks and responsibilities outside their scope of practice. This raised liability issues and concerns about their own well-being. Community health workers also functioned in isolation from the MDPs and other PHC services, resulting in the fragmentation of services. The program included defining and clarifying the CHWs’ roles and establishing referral systems to MDPs and PHC services. Wider service problems were identified, mostly related to the lack of clinical practice pathways, such as delayed referral to HCBC, fragmentation of services and inadequate provision of assistive products. These problems were escalated to service managers, as this fell outside the scope of the educational initiative.

### Design

The design of the training program was based on the information generated during the analysis phase.

#### Program structure and time allocation

The final structure based on the key programmatic outcomes is summarised in Table 4. While there was good consensus on the structure, suggested time allocations varied widely between CIG members. Individuals who regularly provided skills training allowed more time for observation and practice, whereas others allowed more time for theory. The final time allocation for the program was 21 h.

**TABLE 4:** Program outline and time allocation.

Session name and number	Duration in minutes
1. Introduction	15
2. What is a stroke?	55
3. Stroke services and rehabilitation	55
4. Communication problems	55
5. Emotional and social well-being	180
6. Problems with the mind and behaviour	60
7. Positioning	120
8. Moving in bed	180
9. Transfers	180
10. Bladder and bowel management and using the toilet	30
11. Eating, drinking and swallowing safely	45
12. Mouth care	25
13. Washing	50
14. Dressing	30
15. Moving around	120
16. Rehabilitation exercises	60

**Total time**	**1260**

#### Learning outcomes

Through multiple reviews, 15 key programmatic learning outcomes as well as detailed learning outcomes for each of the sessions were identified (Table 5).

**TABLE 5:** Key programmatic outcomes (aims) and learning outcomes of the 16 sessions.

Session	Programmatic learning outcomes	Sessional learning outcomes
**Introduction**		
Introduction	To introduce the trainers and CHWs to each other and to explain the learning outcomes of the training program	By the end of the session, the CHWs should: know the outcomes of the training program have an overview of the timetable have stated their expectations and problem areas agree on the housekeeping rules.
**Stroke information and services**		
What is a stroke?	To enable CHWs to explain to patients who had a stroke and their family caregivers the causes, symptoms≈and problems associated with a stroke, as well as recovery after a stroke	By the end of the session, the CHWs should be able to explain to the patient and the family caregivers: what a stroke is the two main causes of a stroke the risk factors for stroke how to know that someone is having a stroke the symptoms and problems of a stroke recovery after stroke how to minimise the risk for another stroke.
Stroke rehabilitation and services	To enable CHWs to explain the aims, benefits and principles of stroke rehabilitation to patients who had a stroke and their family caregivers with specific reference to the home-based rehabilitation that patients will receive at primary health care level	By the end of the session, the CHWs should be able to explain to the patient and the family caregivers: what rehabilitation is what rehabilitation opportunities are available to patients in their area the aims of a home-based rehabilitation program at primary level the role of the CHWs in home-based stroke rehabilitation what the responsibility of the patient, family and family caregiver is in the home-based rehabilitation program. In addition, they should be able to: know their own limitations and know when to ask for help know how to refer patients to appropriate disciplines or professionals.
**Psychosocial management**		
Emotional and social well-being	To establish and maintain healthy supportive relationships between the CHW, patients who had a stroke and their families and to identify psychosocial risks to the patient’s well-being	By the end of the session, the CHWs should be able to: acknowledge the impact of the stroke on the patients and their families, including trauma and feelings of loss empathise and provide appropriate support to patients and their families recognise the environments, practices and relationships that may be harmful to patients and make the necessary referrals recognise caregiver strain and burnout and support the family caregiver maintain boundaries and know when to request guidance and assistance for themselves (‘care of the caregiver’) make a referral to a social worker.
**Rehabilitation knowledge and skills**		
Communication problems	To enable CHWs to guide and support family caregivers to communicate effectively with patients who had a stroke and who experience problems with communication	By the end of the session, the CHWs should be able to explain and provide guidance to the patient and the family caregivers on: common communication problems how to implement simple strategies to improve communication and understanding how to communicate respectfully with patients how to include the patient in decisions on his or her care. In addition, the CHWs should be able to: make appropriate referrals to a SLT support the home program prescribed by the SLT to improve communication.
Problems with the mind and behaviour	To enable CHWs to teach family caregivers to manage and interact effectively with patients who have had a stroke and who are experiencing problems with the mind (cognitive problems) and behaviour	By the end of the session, the CHWs should be able to teach and provide guidance to family caregivers on: common problems of the mind (cognitive problems) and behaviour how to implement simple strategies to manage these problems how to effectively interact with patients who have problems of the mind (cognitive problems) and behaviour. In addition, by the end of the session the CHWs should be able to: make an appropriate referral to an occupational therapist make an appropriate referral to a social worker.
Positioning	To enable CHWs to guide and support patients who had a stroke and their family caregivers on how to position and support the patients in bed and in a chair	By the end of the session, the CHWs should be able to explain and/or demonstrate to the patient and family caregiver: the benefits of good positioning which positions and/or postures to avoid how to care for the shoulder how to position and support the patient in bed whether lying on the weak side, lying on the strong side, lying on the back or sitting in bed how to position and support the patients when sitting in a chair with or without arm rests how to position and support patients when sitting in a wheelchair, including safety aspects, how to adjust footplate height and the correct use of a wheelchair cushion how to prevent the patient from sliding when sitting how to make low-cost assistive products (lap table and tray table) how to find a solution if the bed is too soft.
Moving in bed	To enable CHWs to guide and support patients who had a stroke and their family caregivers on how to move the patient in bed and how to help the patient move in bed	By the end of the session, the CHWs should be able to explain and demonstrate to the patient and family caregiver: how to help or teach the patient to move up and down in bed; move over to the side or middle of the bed; roll over; and sit up over the side of the bed how to provide appropriate support to help the person move how to protect the patient’s weak shoulder what movements the patient should avoid how to prevent injury of the caregiver. In addition, by the end of the session, the CHWs should be able to: identify when to refer the patient to a physiotherapist make a referral to the physiotherapist.
Transfers	To enable CHWs to guide and support family caregivers on how to transfer or help transfer patients who had a stroke	By the end of the session, the CHWs should be able to explain and demonstrate to the patient and family caregiver: how to support and transfer the patient using lifting transfers, standing transfers and sideways transfers how to support and transfer patients who are fully dependent and need two people’s assistance, need moderate assistance from one person, need little assistance from one person and need standby assistance only how to protect the patient’s weak shoulder what movements the patient should avoid how to prevent injury of the caregiver how to use a transfer board, remove armrests and flip up and swing away footrests how to find a solution if the bed or chair is too low how to do car transfers, including transferring the patient into a car and minibus taxi, correctly folding and opening a wheelchair and safely loading the wheelchair into a vehicle. In addition, by the end of the session, the CHWs should be able to: identify when a foot and ankle splint (ankle foot orthosis [AFO]) is needed and make a referral make appropriate referrals to a physiotherapist or occupational therapist.
Incontinence and toileting	To enable CHWs to guide and support family caregivers to improve bladder and bowel management, do safe toilet transfers and select alternative toileting options for the home bedroom	By the end of the session, the CHWs should be able to: teach family caregivers strategies for bladder and bowel management explain and demonstrate to the patient and family caregiver how to do safe toilet transfers, including where to provide support to help the patient move; how to protect the patient’s weak shoulder; what movements the patient should avoid; and how to prevent injury of the caregiver suggest alternative toileting options and assistive products that can be used in a bedroom make appropriate referrals to a professional nurse and/or doctor.
Eating, drinking, and swallowing	To enable CHWs to guide and support family caregivers to help patients who had a stroke and have difficulty eating, drinking and swallowing to do so safely	By the end of the session, the CHWs should be able to: list common eating, drinking and swallowing problems in patients who had a stroke. In addition, the CHWs should be able to explain and demonstrate to the patient and family caregiver how to: correctly position the patient for eating and drinking safely help patients when eating and drinking, including supporting the head, supporting the jaw and lips to help close the mouth and correctly positioning the spoon or fork and glass or cup identify the need for assistive products to support independent eating and drinking provide the correct fluid and food consistency and texture to serve the patient make appropriate referrals to a SLT, dietician or occupational therapist.
Mouth care	To enable CHWs to teach family caregivers to promote and ensure good mouth and dental care in patients who had a stroke	By the end of the session, the CHWs should be able to: explain and demonstrate to the patient and family caregiver how to clear the mouth of leftover food; guide patients to effectively and safely brush their teeth and rinse their mouth; and clean patients’ mouths when they are unconscious or have low levels of consciousness recommend assistive products to make it easier for patients to hold a toothbrush or reach their mouth make appropriate referrals to an oral hygienist.
Washing	To enable CHWs to teach family caregivers how to wash a patient who had a stroke and is unable to wash themselves and how to help patients to wash themselves	By the end of the session, the CHWs should be able to explain and demonstrate to the patient and family caregiver: how to help the patient move in bed for a bed wash how to prevent injury to the weak shoulder how to set up the environment to enable someone with poor balance to wash or be washed safely while sitting how to wash hard-to-reach areas with one hand how to do bath or shower transfers what assistive products can help with washing how to make low-cost assistive products for washing. In addition, the CHWs should be able to make appropriate referrals to a physiotherapist and/or occupational therapist.
Dressing	To enable the CHW to teach the family caregivers to safely dress the patients who had a stroke and to teach patients how to dress themselves	By the end of the session the CHWs should be able to guide the patient and family caregiver on: the principles of dressing when one side is weak or paralysed how to prevent injury to the weak shoulder clothing to wear that makes dressing easier how to dress when lying down how to dress in a sitting or standing position how to put on foot splints and shoes how to tie shoelaces with one hand. In addition, the CHWs should be able to make appropriate referrals to physiotherapists and occupational therapists.
Moving around	To enable CHWs to teach family caregivers how to help a patient who had a stroke move around at home in a wheelchair or walking	By the end of the session, the CHWs should be able to: explain and demonstrate to the caregiver how to safely push someone in a wheelchair on level terrain; up and down inclines (slopes); and up and down stairs explain and demonstrate to the patient and caregiver how to propel the wheelchair using one hand and one foot; safely help a patient walk; safely help a patient negotiate a step or steps; and get a patient up from the floor after a fall identify barriers to safe mobility and make suggestions for change identify assistive products to improve safety when walking make appropriate referrals to the physiotherapist.
Rehabilitation exercises	To enable CHWs to guide patients who had a stroke and their caregivers on how to do basic rehabilitation exercises	By the end of the session the CHWs should be able to: explain how good posture and correct transfers and handling of the patient are part of rehabilitation management (24-h management) teach the patient and family caregiver basic rehabilitation exercises monitor that exercises are correctly performed advise on the risks of certain popular exercises.

CHW, community health workers; SLT, speech and language therapist.

#### Approach to teaching

To be appropriate for CHWs, the content needed to be aligned with level three of the SA National Qualifications Framework (South African Qualifiations Authority 2012). Principles of adult learning were adopted (Hanger & Wilkinson 2001; Kaufman 2003; Knowles 1970). This allowed for a facilitative style of teaching and active engagement through demonstrations, practice and role-plays in groups of three, with CHWs rotating through three roles: (1) being the stroke survivor, (2) being the family caregiver and (3) being the CHW. Assuming these different roles gave them a vicarious experience of being the recipient of their intervention as well as the CHW. The teaching approach incorporated a spiral curriculum (Harden & Stamper 1999) and a triad of theory–modelling–practice with feedback for learning practical skills (Hanger & Wilkinson 2001; Kaufman 2003; Maguire & Pitceathly 2002).

Considering the practical nature of the training, a ratio of one trainer to six learners was needed for small group work and learning skills. Depending on the size of the training venue, resources and trainers, up to 24 learners could be accommodated in a session.

#### Rehabilitation philosophy underpinning the training program

The Bobath approach (Michielsen et al. 2019; Vaughan-Graham et al. 2009, 2019a, 2019b) was deemed the most appropriate as it follows a functional, problem-solving approach. Recovery is founded on neuromuscular plasticity. By incorporating the principles of task-based training, motor learning and a 24 h approach, normal movement patterns are facilitated and compensatory patterns are limited. This is the only approach to have formally included other categories of healthcare personnel in its training (Friedhoff & Schieberle 2007).

#### Training resources required

The following training resources were required:

- Trainer’s manual with timetable and detailed session plans inclusive of learning outcomes, list of resources needed, guidelines on further adaptation to the local context, preparation required and detailed session plan including timing, teaching methods and approach to formative assessment with model answers and checklists.
- PowerPoint presentations (Microsoft Corporation, Redmond, Washington, United States) to support the theory and process of each session.
- Equipment for demonstration and practice purposes. A standard list of equipment would enable training in different settings, such as a nongovernment organisation, local health care facility or community centre. Although access to tables, chairs, therapy mats and examples of wheelchairs and assistive products would be readily available in most settings, it was unlikely that there would be enough beds or treatment plinths. Therapy mats could be used for practising bed mobility but not for bed or bath transfer training. The training manual had to guide trainers to use chairs and tables to allow simulated transfer practice. Examples of self-made assistive products were also needed.
- Learner’s manual for CHWs based on the content of the trainer’s manual, incorporating the formative assessments and model answers or checklists, as well as resources such as examples of referral forms.
- Detailed sequences of line drawings were needed to supplement the text of the learner’s manual. These same drawings would be used in the other resources as well.
- Booklet for caregivers and stroke survivors. A suitable caregiver and patient booklet was previously co-authored by the researcher and revised after a suitability study (Botha 2008; Scheffler & Visagie 2011) for use in a similar community. The booklet was updated and revised accordingly and translated into Afrikaans and isiXhosa (Stellenbosch University 2022).

#### Assessment

Formative assessments were integrated in each session through case studies and role play activities. Each of the case studies had model answers listed in both trainer’s and learner’s manuals and a checklist for performing activities. These assessments would assist both the trainers and the CHWs to provide or obtain constructive feedback on learning.

### Develop

Line drawings were commissioned from purpose-specific photographs taken by the researcher. The text for the trainer’s manual was developed first, and then the PowerPoints and learner’s manual were developed. The stroke survivor and caregiver information booklet were updated and translated. Training resources were developed in English using plain language. This challenged the MDPs. The learner’s manual and information booklet were picture based with limited text. The manuals and information booklet were developed as both hardcopy and online resources, together with the PowerPoint presentations.

## Discussion

This study appears to be the first of its kind to design an appropriate stroke training program for CHWs to empower caregivers and stroke survivors in the home. Several stroke interventions, including caregiver training, have been developed through co-creation (Aslani et al. 2016; Dobe et al. 2022; Krieger et al. 2021). However, these programs focus on stroke survivors who have received formal rehabilitation services. These programs are primarily delivered by professionals and also include applications and other technological solutions. In this study, the stroke survivors had not received any formal rehabilitation services.

The training program was designed under the PHC philosophy to address a significant community health problem and was developed through coordinated engagement between local professional and nonprofessional healthcare providers using appropriate technology and resources (Mash et al. 2019; Naidoo, Van Wyk & Joubert 2016; World Health Organization 2008). Technological barriers, such as restrictions on the government computer networks, prevented the use of cloud-based collaborative environments during the design and development phase and impacted the participative process. Primary healthcare in SA is evolving, and MDPs in this study had neither training nor experience in developing community-based interventions and had limited time to participate in such initiatives because of their focus on individual care. Participating in this novel process should lay a foundation for future team-based interprofessional community-oriented PHC interventions. Effective leadership and management are needed to reshape existing care models into comprehensive PHC interventions (Marcus, Hugo & Jinabhai 2017; Schneider et al. 2015).

While stroke caregiver training is routinely delivered by rehabilitation professionals (Bakas et al. 2015; Camak 2015; Chaiyawat & Kulkantrakorn 2012; Forster et al. 2015; Hankey 2013; Mudzi et al. 2012; Sabariego et al. 2012; The ATTEND Collaborative Group 2017; Wang et al. 2015), task-shifting to other healthcare cadres is advocated where numbers of rehabilitation professionals are limited (Bryer 2009; Bryer et al. 2011; Hassan et al. 2012; Miranda et al. 2017; The ATTEND Collaborative Group 2017; Wasserman, De Villiers & Bryer 2009). Task-shifting to nurses is common (Deyhoul et al. 2020; Torres-Arreola et al. 2009; Yan et al. 2016; Zhou et al. 2019). However, problems such as increased workload, time implications and inadequate underlying skills and knowledge may undermine such task-shifting (Torres-Arreola et al. 2009; Zhou et al. 2019). No evidence of task-shifting to mid- or grassroots-level workers was found for stroke rehabilitation in LMICs.

Unlike many caregiver training interventions in LMICs, which focus on home-based rehabilitation exercises (Chaiyawat et al. 2009; The ATTEND Collaborative Group 2017; Torres-Arreola et al. 2009; Wang et al. 2015; Yan et al. 2016; Zhou et al. 2019), this training program included minimal teaching of home exercises to ensure that the content was aligned with the CHWs’ scope of practice and avoided overlap with the scope of future midlevel rehabilitation workers. By following the principles of the Bobath neurorehabilitation approach, recovery was promoted through a 24 h therapeutically structured caregiving approach and neuroplasticity by repeated task practice and motor learning. Routinely repeated caregiving activities become therapeutic and synchronous with the therapeutic approach from therapists (Michielsen et al. 2019; Vaughan-Graham et al. 2009, 2019a, 2019b).

The content of this training program was based on an in-depth analysis of the local context and the needs of the caregivers and stroke survivors. In contrast, most caregiver training programs, including those developed in LMICs (Chaiyawat et al. 2009; Chaiyawat & Kulkantrakorn 2012; Deyhoul et al. 2020; Mudzi et al. 2012; Pitthayapong et al. 2017; The ATTEND Collaborative Group 2017; Torres-Arreola et al. 2009; Yan et al. 2016; Zhou et al. 2019), have followed a top-down approach with design by professionals from specialist rehabilitation services. Only a few training programs (Dobe et al. 2022; Krieger et al. 2017; Robinson et al. 2005) have been informed by the needs of the target population.

Although the topics covered in this training program were similar to others in the immediate post-acute period in both high-income countries (HICs) (Bakas et al. 2014; Forster et al. 2015; Kalra et al. 2004; White, Cantu & Trevino 2015) and LMICs (Chaiyawat et al. 2009; Chaiyawat & Kulkantrakorn 2012; Deyhoul et al. 2020; Mudzi et al. 2012; Pitthayapong et al. 2017), it differed from existing programs by focusing on culturally and contextually appropriate information and training materials, including low-cost, self-made assistive products. Each lesson plan also provided guidance on how to further adapt the training to the local setting. Contextual appropriateness has only recently been emphasised in stroke training (Krieger et al. 2017; The ATTEND Collaborative Group 2017; Yan et al. 2016; Zhou et al. 2019).

The low educational levels of both the CHWs and the final target population impacted the selection of teaching methods and the development of training resources. The interactive nature of the adult education teaching model (Hanger & Wilkinson 2001; Kaufman 2003; Knowles 1970) accommodated the low literacy levels (Doak, Doak & Root 1996), different learning styles (Hanger & Wilkinson 2001; Knowles 1970) and learning of practical skills (Hanger & Wilkinson 2001; Kaufman 2003; Maguire & Pitceathly 2002; Mudzi et al. 2012; Nadar & McDowd 2008; Pitthayapong et al. 2017), as well as feedback through assessment (Deyhoul et al. 2020; Hanger & Wilkinson 2001; Kaufman 2003; Maguire & Pitceathly 2002; Mudzi et al. 2012; Pitthayapong et al. 2017). The plain language text of the resources and trainer’s manual and the picture-based format of the learner’s manual and information booklet facilitated access and understanding (Doak et al. 1996).

Poverty, technological barriers (Opoku, Stephani & Quentin 2017) and prohibitive data costs (Independent Communications Authority of South Africa 2017) limited opportunities for CHWs and caregivers to access online information and/or to use electronic teaching and information platforms, necessitating the development of paper-based resources. All resources were also made available online, with online size minimised by using black and white line drawings and low-resolution videos and slide presentations.

With limited access to and availability of psychosocial services, this training program included a substantial focus on psychosocial support within the CHW’s scope of practice, including identification and referral of at-risk families. Although common in training programs in HICs, psychosocial support is less frequent in training programs in LMICs (Pitthayapong et al. 2017; Yan et al. 2016; Zhou et al. 2019).

Although the structure of the training program for CHWs followed a specific sequence and duration, the training of caregivers would be tailored to the stroke survivor’s specific level of functioning and care needs, similar to most training programs (Chaiyawat et al. 2009; Chaiyawat & Kulkantrakorn 2012; Forster et al. 2015; McLennon et al. 2014; Mudzi et al. 2012; Pitthayapong et al. 2017; The ATTEND Collaborative Group 2017; Wang et al. 2015; Yan et al. 2016; Zhou et al. 2019). Progression of caregiver training would be determined by the care coordinators and the CHWs.

As a participatory action research project, the involvement of the service providers was limited because of low numbers and high service demands. None had experience in designing a training program of this scale and nature. Fully collegial roles (Higginbottom & Liamputtong 2015) with equal responsibility for conducting the research project and compiling and implementing the training program were not possible. Cooperative inquiry group members therefore clarified the extent of their involvement at the start. Whereas CIG members are usually both co-researchers and co-subjects during the inquiry (Heron 2014), limited availability shifted the focus to the pragmatic tasks of developing the training program, with reflectivity limited to more practical and operational awareness. Transferability and use of the training program will be limited to similar services, contexts and group characteristics and will need to be adapted for the local context with regard to resources, technology, health care service structure and existing knowledge. The design of the resources allows for this. This article only reports on the planning phase of the inquiry. A future article will report on the implementation of the program and subsequent actions, observations and reflections of the CIGs.

## Conclusion

This study demonstrated how local healthcare services at the PHC level can design an appropriate, contextually relevant community-oriented intervention through a participatory approach. The CI planning step described in this article used the ADDIE model to analyse, design and develop an appropriate home-based stroke rehabilitation program to be delivered by CHWs in a low-resourced setting. This home-based stroke rehabilitation program and its accompanying training program for CHWs should be implemented and further evaluated in practice.

## Footnotes

1 Community health workers are selected from and work as part of ward-based primary health care outreach teams in the communities in which they live. Standardised national accredited training only started in 2016. Many existing CHWs have limited training. Their scope includes health promotion and illness prevention; referral to health services; adherence support and counselling for those with chronic conditions; curative services; and home-based care, which includes facilitation of daily living activities and restoring function, as well as providing psychosocial support.

2 Midlevel rehabilitation workers work within facilities and community-based rehabilitation services and include physiotherapy and occupational therapy technicians and rehabilitation care workers. Their training programs are of longer duration and at higher educational level than those of CHWs.
